# Transfection of Arctic *Bryum* sp. KMR5045 as a Model for Genetic Engineering of Cold-Tolerant Mosses

**DOI:** 10.3389/fpls.2020.609847

**Published:** 2021-01-08

**Authors:** Mi Young Byun, Suyeon Seo, Jungeun Lee, Yo-Han Yoo, Hyoungseok Lee

**Affiliations:** ^1^Division of Life Sciences, Korea Polar Research Institute, Incheon, South Korea; ^2^Polar Science, University of Science and Technology, Incheon, South Korea

**Keywords:** Arctic moss, axenic culture, *Bryum* sp., protoplast, transfection

## Abstract

Mosses number about 13,000 species and are an important resource for the study of the plant evolution that occurred during terrestrial colonization by plants. Recently, the physiological and metabolic characteristics that distinguish mosses from terrestrial plants have received attention. In the Arctic, in particular, mosses developed their own distinct physiological features to adapt to the harsh environment. However, little is known about the molecular mechanisms by which Arctic mosses survive in extreme environments due to the lack of basic knowledge and tools such as genome sequences and genetic transfection methods. In this study, we report the axenic cultivation and transfection of Arctic *Bryum* sp. KMR5045, as a model for bioengineering of Arctic mosses. We also found that the inherent low-temperature tolerance of KMR5045 permitted it to maintain slow growth even at 2°C, while the model moss species *Physcomitrium patens* failed to grow at all, implying that KMR5045 is suitable for studies of cold-tolerance mechanisms. To achieve genetic transfection of KMR5045, some steps of the existing protocol for *P. patens* were modified. First, protoplasts were isolated using 1% driselase solution. Second, the appropriate antibiotic was identified and its concentration was optimized for the selection of transfectants. Third, the cell regeneration period before transfer to selection medium was extended to 9 days. As a result, KMR5045 transfectants were successfully obtained and confirmed transfection by detection of intracellular Citrine fluorescence derived from expression of a *pAct5:Citrine* transgene construct. This is the first report regarding the establishment of a genetic transfection method for an Arctic moss species belonging to the Bryaceae. The results of this study will contribute to understanding the function of genes involved in environmental adaptation and to application for production of useful metabolites derived from stress-tolerant mosses.

## Introduction

Bryophytes include about 20,000 species across three phyla of the kingdom Plantae. Mosses (phylum Bryophyta) are the most abundant of the three bryophyte groups and the second largest (over 13,000 species) next to phylum Tracheophyta (about 345,000 species) in the number of extant species. Liverworts (phylum Marchantiophyta) account for approximately 7,200 species, and the hornwort (phylum Anthocerotophyta) group numbers about 220 species (Roskov et al., 2019). Mosses are evolutionarily located intermediate between single-celled algae and complex seed plants, which provides a significant opportunity to investigate the evolution of a variety of plant traits (Prigge and Bezanilla, 2010). Although the study of mosses has a long history (Reski, 1998), most studies have focused on the model species *Physcomitrium patens* (previously known as *Physcomitrella patens*; Medina et al., 2019). Among several features of *P. patens* that made it suitable as a model moss species, the development of an easy and efficient transformation method for *P. patens* was the most important advance for research on the fundamental biology and biotechnology of mosses (Schaefer et al., 1991). Furthermore, with the release of the complete *P. patens* genome sequence (Rensing et al., 2008), precise and efficient genetic manipulation to explore biological functions of genes responsible for the unique features of the moss has become possible (Cove et al., 2009a). Subsequently, several studies have reported the development of powerful bioengineering tools for the production of high-value compounds in mosses, such as terpenoids, biopharmaceuticals, and cosmetic products (Bach et al., 2014; Reski et al., 2015; Wandrey et al., 2018).

Until recently, only two species of mosses other than *P. patens* could be genetically manipulated by PEG-mediated protoplast transformation (Thümmler et al., 1992; Trouiller et al., 2007; Nomura et al., 2016). *Ceratodon purpureus* is a moss species used as a model system for genetic studies (Wagner et al., 1997). Genetic transformation of *C. purpureus* was demonstrated by the transgenic expression of the oat phytochrome gene (*phyA*) (Thümmler et al., 1992), and the efficiency of gene targeting was compared to that of *P. patens* (Trouiller et al., 2007). A recent study showed efficient and heritable targeted mutagenesis using the CRISPR/Cas9 system in the non-model moss *Scopelophila cataractae* (Nomura et al., 2016). Despite pioneering attempts to establish ways to modify the genetic information of non-model mosses, genetic modification methods still lag far behind those available for flowering plants or *P. patens* due to the distinctive cellular and/or molecular properties of moss species.

The Arctic is a polar region located at the northernmost region of the Earth and the climate is characterized by freezing winters and cold summers. Therefore, plants grow relatively close to the ground and form tundra consisting of dwarf trees, herbaceous plants, lichens, and mosses (Walker et al., 2016). Since mosses possess a variety of features that are adaptive to the Arctic environment, such as high poikilohydry, pluripotency, and cold/freezing tolerance, they contribute substantially to the vegetative biomass and the species richness of vegetation in the Arctic region (Oechel and Sveinbjörnsson, 1978; Lewis et al., 2017). More than 380 bryophyte species have been recorded from the Svalbard Archipelago, which greatly exceeds the more than 175 species of vascular plants found there. Mosses may function to promote the settlement of vascular plants by conserving soil moisture and reducing soil temperature fluctuations in early successional stages of barren land (Prestø et al., 2014). Bryophytes occasionally show variations among populations, indicating distinct responses to unfavorable environmental stimuli and adaptive attributes in physiological and morphological traits representing subspecies, variants, or forms (Longton, 1988; Meltofte, 2013). Phenotypic plasticity is one of the most prominent characteristics enabling bryophytes to fit into and colonize polar environments and confers considerable tolerance to dehydration and frost stress (La Farge et al., 2013; Stanton and Reeb, 2016). Several studies have reported metabolic and physiological characterization of Arctic moss. The photosynthetic CO_2_ fluxes for two sub-Arctic moss species, *Polytrichum piliferum* and *Sphagnum fuscum*, exhibited seasonal changes in photosynthetic capacity which were important in determining total gross primary productivity (Street et al., 2013). Arroniz-Crespo et al. (2011) showed that the effects of increased UV-B radiation on three Arctic bryophytes were dependent on their plasticity. Due to recent advances in next-generation sequencing, the *Sphagnum* microbiome has revealed characteristics of the microbiome community composition in peat mosses (Kostka et al., 2016). In addition, molecular phylogenetic analysis implied that DNA barcoding with a combination of several markers improves species identification of Arctic mosses (Lang et al., 2014). However, the knowledge of Arctic bryophytes remains in its infancy. Furthermore, the lack of genetic transfection of Arctic mosses has limited research into their distinctive traits.

In this study, we selected the Arctic moss *Bryum* sp. KMR5045 as a research target. The most well-known species in the genus *Bryum* is *B. argenteum*. Due to the nature of its cosmopolitan distribution in a variety of climates, including dry, hot, medium and cold habitats, various studies have been reported, including the dessication tolerance for 13 ecotypes (Greenwood et al., 2019), the establishment of *in vitro* culture conditions from spores (Sabovljević et al., 2005) and the effect of plant hormones on *in vitro* development (Sabovljević et al., 2010). Also, it has also been previously reported on the culture conditions for protonema and gemetophore of *B. coronatum*, which is known to contain various phytochemicals (Pandey et al., 2014). However, there have been no reports of *in vitro* cultivation for Arctic *Bryum* species, and no transfection studies have been reported for species in the genus *Bryum*. As the first step to building a model system for studying unique features of Arctic mosses, a protonema-derived suspension culture of KMR5045 was established. In addition, a genetic transfection protocol was developed by adapting the *P. patens* protocol to KMR5045. Genetic engineering of this moss might serve multiple purposes, such as identifying molecular mechanisms of environmental adaptation of mosses and production of biological products using bryophytes as a platform for molecular farming.

## Materials and Methods

### Plant Collection and Culture

*Bryum* sp. KMR5045 plants growing under natural conditions were collected in the vicinity of the Arctic Dasan station (78°55′ N; 11°56′ E) on Spitsbergen Island of the Svalbard Archipelago of Norway in July 2014. A dried specimen was deposited in the KOPRI Herbarium^1^ in Korea with the number KOPRI-MO00900. Dried gametophores were washed with distilled water and subsequently sterilized with 0.2% NaClO solution. Sterilized tissue was placed on solid Knop medium (Schween et al., 2003). Surface-sterilized gametophore-derived protonema tissue was subcultured using a T-10 basic ULTRA-TURRAX disperser (IKA, Germany) and transferred to new medium at 3-week intervals. Tissue was grown in a growth chamber at 20°C under a 16/8-h photoperiod.

### Phylogenetic Analysis

Total genomic DNA (gDNA) was extracted from the protonema of KMR5045 using cetyl trimethylammonium bromide (CTAB) buffer (2% CTAB, 2% PVP-40, 20 mM EDTA, 1.4 M NaCl, and 100 mM Tris HCl, pH 8.0). Extracted gDNA was used as a template for PCR. PCR analysis was performed in 20 μL reactions containing 1 μL of gDNA template, 1 μM of each primer, and 10 μL of EmeraldAmp GT PCR Master Mix (TaKaRa, Japan). The amplification procedure was as follows: 10 min of denaturation and enzyme activation at 95°C followed by 30 cycles at 95°C for 30 s, 55°C for 30 s, and 72°C for 30 s. The DNA sequences of the primers used for *rps4* and *trn*L-F amplification are listed in Supplementary Table 1. To construct a phylogenetic tree including KMR5045, *rps4* + *trn*L-F combined nucleotide sequences were compared with those of other species obtained from GenBank (Supplementary Table 2) (Cox et al., 2000). Sequences were aligned using the MAFFT program (Katoh and Standley, 2013) and downstream analyses were performed using MEGA X software (Kumar et al., 2018). The phylogenetic tree was constructed from the datasets using neighbor-joining analysis and evolutionary distances were computed using the Jones-Thornton-Taylor (JTT) matrix-based method. Supports for internal branches were tested using bootstrap analyses of 1,000 replications.

### Protoplast Isolation

Seven-day-old protonema tissues of KMR5045 were used to isolate protoplasts. Protonema were pre-incubated for 1 h in 0.5 M mannitol with slow rotation. Two experiments were performed to optimize the mannitol buffer and enzyme concentrations. The first experiment was designed to determine the optimal concentration of mannitol buffer. Three mannitol concentrations (0.4, 0.5, and 0.6 M) were tested in the isolation procedure. The second experiment was designed to compare the effect of enzyme concentration on protoplast yield and viability. Three concentrations (0.5, 1.0, and 2.0%) of driselase (Sigma-Aldrich, United States) dissolved in 0.5 M mannitol were tested. After induction of cell plasmolysis with mannitol, the cell suspension was treated with enzyme solution and incubated in darkness for 2 h at 20°C with constant gentle agitation on a rotary shaker. The digested cells were passed sequentially through 100 and 40 μm nylon sieves and the filtrate was centrifuged in a swinging-bucket rotor (124 × *g*, 10 min). The pellet was resuspended in 20 mL of 0.5 M mannitol and centrifuged again. After centrifugation, the pellet was resuspended in 0.5 M mannitol. The isolation yield of protoplast cells was determined using a counting chamber under a visible light Axio Imager A2 microscope (Carl Zeiss, Germany). The experiments were conducted with four biological replications.

### Antibiotic Treatment

The effects of antibiotics, hygromycin (AG scientific, United States), G418 (Roche, United States), and kanamycin (Sigma-Aldrich) on cell growth were tested for both protonema tissues and protoplast cells. To compare sensitivities to antibiotics, KMR5045 and *P. patens* protonema tissues were subcultured onto solid medium containing antibiotics. After 2 weeks of treatment, protonemal growth was assessed and photographed. To test the effect of antibiotics on the growth and development of protoplast cells, cells overlaid onto cellophane were transferred to solid medium containing antibiotics and incubated for 2 weeks at 20°C, and then transferred to antibiotic-free medium. After two rounds of antibiotic selection, the plates were photographed and the surviving plants were counted. The experiments were conducted with four biological replications.

### PEG -Mediated Transfection

Protoplast transfections were conducted as described previously (Cove et al., 2009a) with modification. Freshly isolated protoplasts were counted and resuspended at 1.2 × 10^6^ mL^–1^ in 3M medium (5 mM MgCl_2_, 0.1% MES, 0.48 M mannitol, pH 5.6). Transfection was performed using a *pAct5:Citrine* construct (Mueller et al., 2014). DNA was prepared using a Plasmid DNA purification kit (Macherey-Nagel, Germany) and linearized with *Ase*I restriction enzyme (NEB, United States). The digested DNA was isolated and purified using a NucleoSpin Gel and PCR Clean-up kit (Macherey-Nagel) and resuspended in 0.1 M Ca(NO_3_)_2_ at a concentration of 500 ng**⋅**μL^–1^. The 50 μg of DNA solution was transferred to a sterile round-bottom tube (SPL Life Science, South Korea) and then 250 μL of the protoplast-suspension was added to the tube and mixed gently, followed by the addition of 350 μL of PEG solution (freshly made 40% w/v PEG4000 dissolved in 3M medium). The mixtures were incubated at room temperature for 30 min with occasional gentle mixing. Subsequently, the solution was diluted at 3-min intervals by adding 3M medium at increasing volumes of 1, 2, 3, and finally 4 mL. Protoplasts were centrifuged (124 × *g*, 10 min), resuspended in regeneration medium [250 mg**⋅**L^–1^ KH_2_PO_4_, 250 mg**⋅**L^–1^ MgSO_4_ ⋅ 7H_2_O, 250 mg**⋅**L^–1^ KCl, 1 g**⋅**L^–1^ Ca(NO_3_)_2_ ⋅ 4H_2_O, 12.5 mg**⋅**L^–1^ FeSO_4_ ⋅ 7 H_2_O, 50 g**⋅**L^–1^ glucose, 30 g**⋅**L^–1^ mannitol, pH 5.8, sterilized through a 0.22 μm filter], transferred to six-well plates, and incubated for 24 h in darkness. Thereafter, regenerating protoplasts were transferred to Knop agar medium overlaid with cellophane. After 9 days, the cellophane overlays were transferred to Knop medium supplemented with 15 mg**⋅**L^–1^ hygromycin. Antibiotic-resistant plants were isolated after a second round of selection. The experiments were conducted with three biological replications.

### Analysis of Transgenic Lines

#### RT-PCR Analysis

Total RNA was extracted from protonema tissue using a MiniBEST Plant RNA Extraction Kit (TaKaRa) according to the manufacturer’s protocol. The quantity and quality of RNAs were determined by spectroscopic analysis using a NanoDrop Lite Spectrophotometer (Thermo Scientific, United States). First-strand cDNA was synthesized from 2 μg of total RNA using TOPscript reverse transcriptase (Enzynomics, South Korea) and oligo (dT) primers. RT-PCR analysis was performed in 20 μL reactions containing 1 μL of cDNA template, 2 μM of each primer, and 10 μL of TB Green Premix Ex Taq. The amplification procedure was as follows: 5 min of denaturation and enzyme activation at 95°C followed by 28 cycles at 95°C for 30 s, 55°C for 30 s, and 72°C for 30 s. The *tubulin* gene was used as an internal control (Gao et al., 2015). The DNA sequences of the primers used for PCR amplification are listed in Supplementary Table 1.

#### Immunoblot Analysis

Protonema of KMR5045 were ground to a fine powder using TissueLyser II (Qiagen, Germany) and suspended in extraction buffer [100 mM HEPES buffer pH 7.5, 1 mM β-mercaptoethanol, and protease inhibitor cocktail (Cat. No. C791Z41; Roche)]. The homogenates were centrifuged at 16,000 × *g* for 15 min at 4°C and 25 μg of total protein was separated by 10% SDS-PAGE and blotted to a PVDF membrane (Millipore, Germany). The membrane was subjected to immuno-blot analysis using anti- green fluorescent protein antibody (Clontech, Japan) or anti-tubulin antibody (Sigma-Aldrich), and then HRP-conjugated anti-mouse IgG (Santa Cruz biotechnology, United States). Anti-GFP antibody (Clontech) was used to detect Citrine because of its high similarity to GFP (Griesbeck et al., 2001). The blot was analyzed using SuperSignal Chemiluminescent Substrates (Thermo Scientific) and LAS-3000 Imaging System chemiluminescence analyzer (Fujifilm, Japan).

#### Confocal Microscopy

Stably expressed Citrine signals in 5-day-old protonema cells were observed using a cooled CCD camera (PCO) by a laser scanning confocal microscope (LSM510 META; Carl Zeiss). The fluorescence signals were measured using a sliding 0.3 μm detection window in a confocal microscope. Citrine fluorescence was excited by a laser at 488 nm and chlorophyll was excited by 550 nm laser (Ahn et al., 2018).

## Results

### Establishment of Axenic Culture of the Arctic Moss KMR5045

**FIGURE 1:**
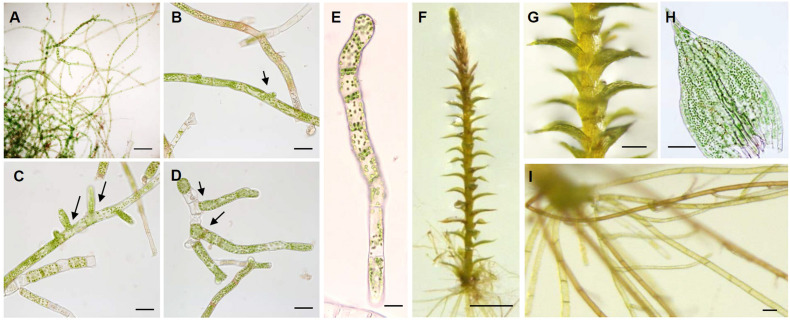
Characterization of axenic cultivation of KMR5045. Morphological analysis of cells in the protonema stage. **(A)** Filamentous protonema cells developed after 5 days of cultivation. **(B–D)** Side branching and filament elongation are indicated by the arrow. **(E)** Chloronema cells composed of rectangular cells with chloroplasts and transverse cross walls. Gametophore stages of KMR5045. **(F)** Gametophore cultivated for 1 month on solid Knop medium. **(G)** Growth of new shoots. **(H)** Leaf structure of KMR5045 gametophore. **(I)** Rhizoids with brown-pigmented walls. Scale bars **(A,G)** 200 μm; **(B–D,H,I)** 50 μm; **(E)** 20 μm; **(F)** 1 mm.

Moss samples were collected from a population growing under natural conditions near the Arctic Dasan station (78°55°56° E), as in *in vitro* cultivation of *Bryum coronatum* protonema (Pandey et al., 2014). Older filaments developed multilayered thickened and often slightly brown-pigmented walls (Figure 1B). In addition, side branches formed from the middle parent cell following filament elongation (Figures 1C,D). Adjacent cells of branching point of the parent filament contained less developed chloroplasts (Supplementary Figure 1), which is a typical feature of caulonema cells of *P. patens* (Reski, 1998). The filaments were made up of short rectangular cells which its length and width of the representative cell was 20 and 40 μm, respectively. In addition, it showed transverse cross walls and contained numerous peripheral ovoid chloroplasts (Figure 1E). Next, we monitored KMR5045 gametophore development on Knop plates. Within 1 month, new shoots were produced from the protonema by apical elongation with copious production of clusters of rhizoids scattered throughout the base of the shoots (Figure 1F). Leaves were equally spaced along the stem (Figure 1G) and broadly rounded and obtuse to acuminate at the apex (Figure 1H). Rhizoids displayed light-yellow to reddish-brown and filaments with oblique septa (Figure 1I) and some points of side-branching were observed (Supplementary Figure 2).

**FIGURE 2:**
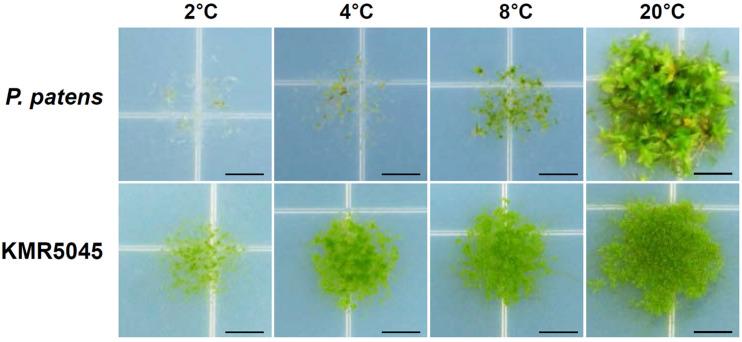
Comparison of growth responses of *P. patens* and KMR5045 under low-temperature conditions. Protonema tissues were inoculated onto fresh Knop medium and then grown at 2, 4, 8, and 20°C for 5 weeks before photographs were taken. Scale bars = 5 mm.

Because KMR5045 was collected from the Arctic region, the growth characteristics of protonema tissues was examined at low temperatures of 2, 4, 8, and 20°C using *P. patens* as a control. Growth of *P. patens* was severely repressed at 8°C relative to growth at 20°C, and stopped almost completely at 2 and 4°C. By contrast, KMR5045 exhibited marked growth at 4 and 8°C and maintained viability even at 2°C (Figure 2). These results imply that KMR5045 has inherent tolerance to low-temperature conditions that enables this species to survive in the Arctic.

### Identification and Molecular Phylogenetic Analysis of KMR5045

**FIGURE 3:**
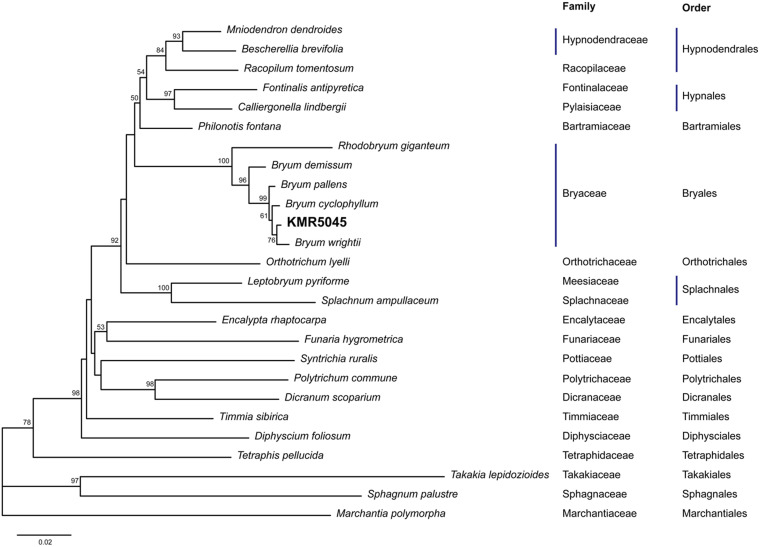
Molecular phylogenetic analysis of KMR5045. Amplified *rps4* and *trn*L-F sequences were aligned with sequences from other mosses and analyzed using the neighbor-joining method. The phylogenetic tree shows that KMR5045 is closely related to *Bryum wrightii*.

Due to recent advances in molecular genetics, several molecular markers are available for mosses (Cox et al., 2000). Two molecular markers of the chloroplast genome (*rps*4 and *trn*L-F) were used to identify KMR5045. The lengths of the sequenced markers were 695 and 544 base pairs for *rps*4 and *trn*L-F, respectively. Corresponding sequences from other species were obtained from GenBank (Supplementary Table 2). In the phylogenetic tree constructed by the neighbor-joining method using *rps4* and *trn*L-F sequences, KMR5045 was most closely related to *Bryum wrightii* with a bootstrap value of 76, and formed the Bryaceae clade with *B. wrightii*, *B. cyclophyllum*, *B. pallens*, *B. demissum*, and *Rhodobryum giganteum*, which was distinct from other bryophyte families (Figure 3).

### Protoplast Isolation and Evaluation of Selectable Markers for Genetic Transfection

**FIGURE 4:**
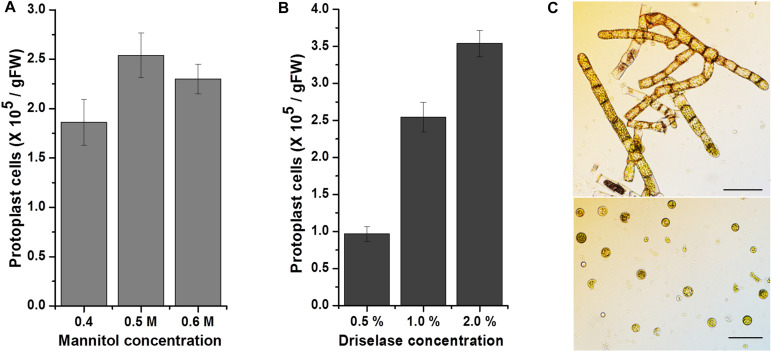
Effects of mannitol and driselase concentrations on KMR5045 protoplast yield. Influence of **(A)** mannitol and **(B)** driselase concentrations on the isolation efficiency of KMR5045 protoplasts. **(C)** Seven-day-old protonema cells (upper panel) and isolated KMR5045 protoplast cells (lower panel). Scale bars (upper panel) 50 μm; (lower panel) 20 μm.

Since most methods for the genetic transfection of mosses are based on isolation of protoplasts and a PEG-mediated gene transfer system (Thümmler et al., 1992; Trouiller et al., 2007; Cove et al., 2009b; Nomura et al., 2016), protoplast isolation was the first step in the effort to transfect KMR5045. We used an aerated liquid culture and induced cell plasmolysis prior to enzymatic cell wall digestion. Mannitol was used for the plasmolysis step and as the buffer in enzyme solution. The concentration of mannitol affects the protoplast isolation yield and the viability of isolated cells (Yu et al., 2017). Therefore, we tested various mannitol concentrations with the enzyme solution (1.0% driselase), and found that 0.5 M mannitol produced the highest protoplast isolation efficiency of 2.5 × 10^5^ cells per gram (Figure 4A). Additionally, the concentration of the enzyme solution also determines the efficiency of protoplast isolation (Duquenne et al., 2007). Therefore, the protoplast isolation yield was compared using various driselase concentrations (Figure 4B). A positive correlation was observed between protoplast yield and the driselase concentration (0.5 and 2.0%). However, the increase in yield was not proportional to the difference in enzyme concentration. Consequently, 1.0% driselase solution dissolved in 0.5 M mannitol in the cell wall digestion reaction was chosen as the optimal combination for KMR5045 protoplast isolation (Figure 4C). When applying these conditions, the regeneration rate (%) of protoplasts of KMR5045 was 16.75 ± 2.9, and that of *P. patens* used as a control was 21.0 ± 4.7 (*n* = 4).

**FIGURE 5:**
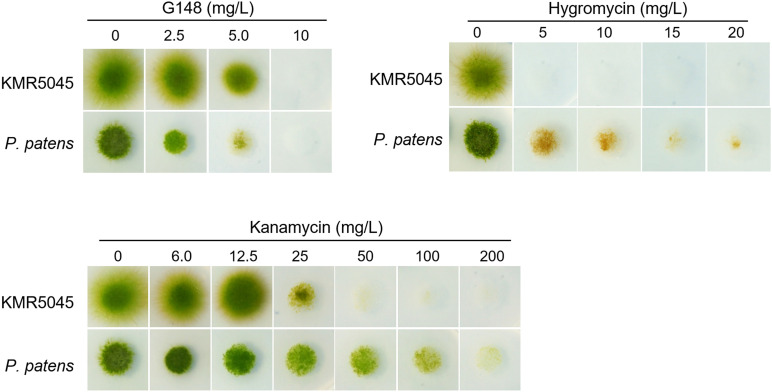
Growth of KMR5045 and *P. patens* protonema cells under various antibiotic concentrations. Scale bars = 200 μm

Several selective agents and corresponding resistance genes have been developed to distinguish transfected and non-transfected cells (Simmonds and Grainger, 1993). The most widely used agents are hygromycin, G418, and kanamycin, which are aminoglycoside compounds that hinder translation in eukaryotic and prokaryotic cells (Mingeot-Leclercq et al., 1999). We determined the sensitivity of KMR5045 protonema cells to various concentrations of antibiotics to determine the suitability of the selection markers for genetic transfection. Cells of *P. patens* cells were also tested for comparison. Subcultured protonema cells were inoculated onto solid Knop medium that was supplemented with various concentrations of antibiotics. The plates were incubated for 2 weeks at 20°C and growth was monitored and compared with that of cells grown on antibiotic-free medium. Both species showed similar levels of sensitivity to G418 and hygromycin, growth of healthy green protonema was notobserved on medium containing >10 mg⋅L^–1^ of G418 or >5 mg⋅L^–1^ of hygromycin. By contrast, the responses of the two mosses to kanamycin differed markedly. While reduced *P. patens* growth was still observed on medium containing 100 mg⋅L^–1^ kanamycin, KMR5045 growth was not detectable on medium containing kanamycin at concentrations of 50 mg⋅L^–1^ or greater (Figure 5).

**FIGURE 6:**
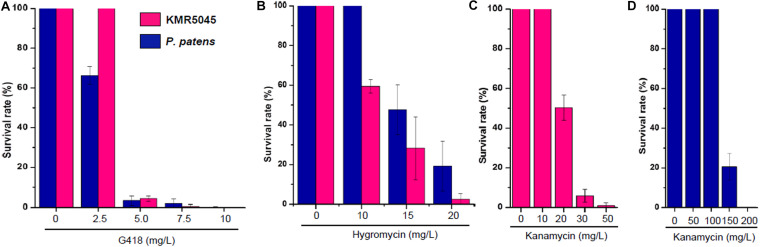
Survival rates of regenerated KMR545 and *P. patens* protoplasts on Knop medium containing various concentrations of antibiotics. **(A)** G418. **(B)** Hygromycin. **(C,D)** Kanamycin.

Next, we determined the sensitivity of KMR5045 protoplasts to various antibiotic concentrations to mimic the regeneration procedure of genetic transfection methods that use protoplast cells. Cellophane overlays carrying protoplasts were transferred to solid Knop medium supplemented with various concentrations of antibiotics. The plates were incubated for 2 weeks at 20°C and then the overlays were transferred to antibiotic-free medium and the number of surviving plants was counted after 2 weeks. As shown in Figure 6, the growth-inhibition effects of the antibiotics on the protoplasts differed between the two species. KMR5045 protoplasts were more resistant to G418 and survived fully at a concentration of 2.5 mg⋅L^–1^, while 66.3% of the *P. patens* cells survived (Figure 6A). By contrast, KMR5045 exhibited a somewhat higher sensitivity to hygromycin, 40.8% of the KMR5045 protoplasts died on 10 mg⋅L^–1^ hygromycin, but all the *P. patens* protoplasts survived under the same condition (Figure 6B). Furthermore, the two species showed quite different responses to kanamycin. KMR5045 displayed high sensitivity to kanamycin with only 1% of survival at concentrations of 50 mg⋅L^–1^ and completely no survival at greater concentration (Figure 6C). By contrast, *P. patens* was more resistant to kanamycin and all protoplasts survived at a concentration of 100 mg⋅L^–1^ and 20.6% survived even at a concentraion of 150 mg⋅L^–1^ (Figure 6D). These results show that the optimal choice of types and concentrations of antibiotics is specific for the moss species targeted for genetic transfection.

### Transfection of Protoplasts via PEG-Mediated Gene Transfer

**FIGURE 7:**
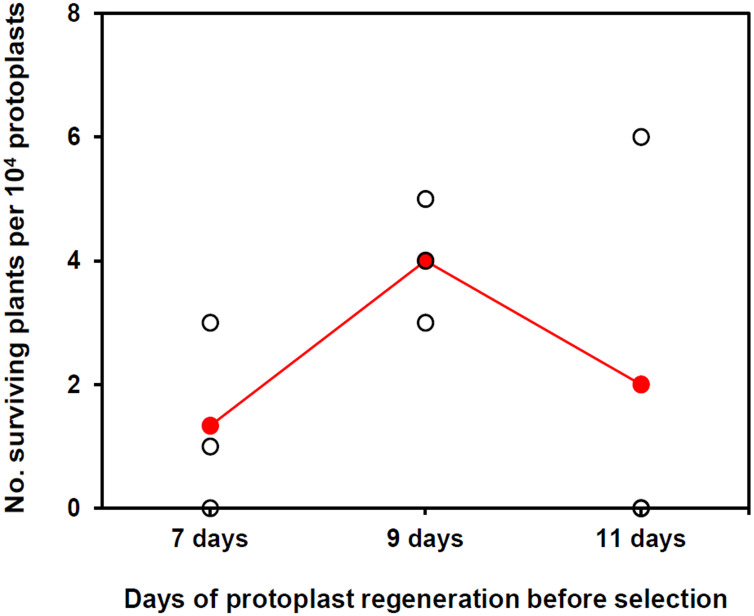
The number of surviving plants after protoplast regeneration for different periods followed by two rounds of hygromycin selection. Colored dots indicate the means of three biological replicates.

To establish a method for PEG-mediated DNA transfer into KMR5045 protoplasts, a construct containing the *Citrine* gene driven by the actin 5 promoter (pAct5) from *P. patens* was used as a reporter (Mueller et al., 2014; Kamisugi et al., 2016). Procedures for the genetic transfection of KMR5045 were modified from those used for *P. patens* (Cove et al., 2009b). The most critical difference was the incubation period for protoplast regeneration before antibiotic selection. In the general process of *P. patens* transfection, protoplasts are transferred to selection medium after a 5-day regeneration period (Cove et al., 2009b). Since KMR5045 protoplasts grow and become visible more slowly than do *P. patens* protoplasts, the regeneration period required modification. To establish the optimal conditions for selection, the cellophane overlay carrying KMR5045 protonema cells was transferred to selection medium containing 15 mg⋅L^–1^ hygromycin after 7, 9, and 11 days of regeneration. Under these conditions, cells showed the highest survival rate and the most distinct selection after regeneration for 9 days (Figure 7). Table 1 shows the detailed information on frequencies of surviving plants depending on days of incubation before transfer to selection medium.

**TABLE 1:** Frequencies of surviving plants in the course of the selection.

Experiments	Frequency^a^ of surviving plants					
	(days before transfer to selection medium)					
	7		9		11	
	1st^b^	2nd^c^	1st	2nd	1st	2nd
1	43	1	154	3	72	6
2	97	0	133	5	32	0
3	115	3	178	4	81	0

*^*a*^Frequency of surviving plants per 10^4^ protoplasts.*

*^*b*^Surviving plants after 1st incubation on selection medium with 15 mg⋅L^–1^ hygromycin.*

*^*c*^Surviving plants after 2nd incubation on selection medium with 15 mg⋅L^–1^ hygromycin.*

### Analysis of Transfectants

**FIGURE 8:**
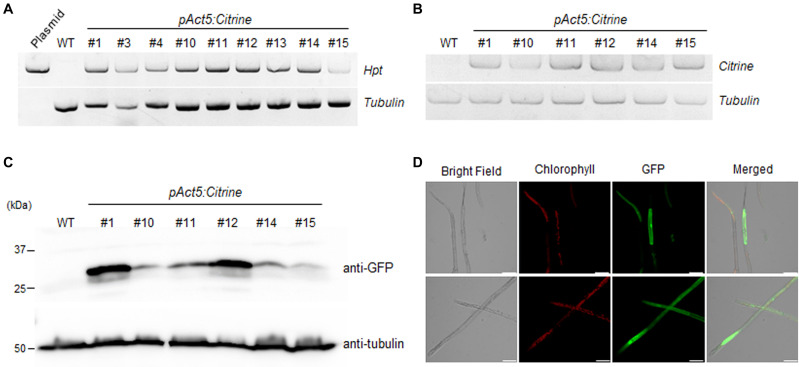
Molecular analysis of *pAct5:Citrine* transgenic moss. **(A)** Genomic PCR analysis of transgenic lines and wild-type (WT) moss. Genomic DNA was extracted from protonema cells and used for PCR to amplify the hygromycin phosphotransferase (*hpt*) gene. **(B)** Reverse transcription (RT)-PCR analysis of *Citrine* expression in six transgenic lines. *Tubulin* was used as an internal control. **(C)** Immunoblot assay. Citrine was detected using anti-GFP antibody. Blot analyzed with anti-tubulin showed equal loading. **(D)** Confocal microscopy. Expression of fluorescent Citrine protein in *pAct5:Citrine* line #1 was visualized by confocal microscopy. Fluorescence was excited by a laser at 488 nm. Scale bars = 50 μm.

Surviving KMR5045 plants were selected after 6 weeks of selection procedure composed of, 2 weeks of incubation on medium with 15 mg L^–1^ hygromycin, 2 weeks of incubation on antibiotic-free medium, and another 2 weeks of incubation on selection medium with 15 mg⋅L^–1^ hygromycin. To examine chromosomal integration of the transgene construct in the protonema tissue of surviving KMR5045 plants, PCR was perfomed to test the presence of the hygromycin phosphotransferase (*hpt*) gene. The *hpt* gene was detected in all transgenic lines tested (Figure 8A). In addition, we assessed *Citrine* transgene expression by semi-quantitative RT-PCR and selected six transgenic lines expressing *Citrine* in the protonemal stage (Figure 8B). Next, the ectopic expression of Citrine protein was examined by immuno-blot assay. Anti-green fluorescent protein (GFP) antibody was used to detect Citrine because of its high similarity to GFP (Griesbeck et al., 2001). As shown in Figure 8C, Citrine protein was detected by anti-GFP antibody in all six transgenic lines, which displayed different levels of expression. Furthermore, florescence signals of the Citrine protein was observed by confocal microscopy in *pAct5:Citrine* line #1, which exhibited the highest level of Citrine protein expression. The florescence signals were diffuse in the cytosol and nucleus as expected (Figure 8D). These results indicate that the transfection method described here can be used to generate KMR5045 transfectants.

## Discussion

As bryophytes are located evolutionarily between algae and terrestrial plants, they provide significant clues about the evolution of plants (Prigge and Bezanilla, 2010). In particular, *P. patens* has been actively studied for the past 20 years and several recent reports have addressed the use of *P. patens* as a platform for the production of high-value compounds (Reski et al., 2018). This elevates the value of studying mosses as a production platform in addition to their value as a research subject for basic science. Efficient transfection and whole-genome sequence information have been indispensable technical advances that have increased the academic and industrial value of *P. patens* (Rensing et al., 2008).

Diverse moss species have colonized the Arctic and Antarctic regions and play a crucial role as primary producers (Street et al., 2013; Walker et al., 2016; Robinson et al., 2018). Study of the adaptive mechanisms of mosses living in extreme environments is essential for the development of useful genetic resources and the investigation of their unique physiological traits. Recent studies on mosses, such as characterization of physiological function and analysis of chloroplasts and mitochondrial genomes, revealed features of extremophytes (Longton, 1988; Pannewitz et al., 2005; Park et al., 2018; Byun et al., 2019); however, the establishment of protocols for the cultivation and genetic transfection of these plants is required for further advances in both basic and applied research.

In this study, we collected the Arctic moss, KMR5045, from the Svalbard Archipelago in the Arctic region and successfully established an axenic culture method. We examined the adaptive characteristics of KMR5045 at low temperatures and found that it maintained its growth and displayed higher tolerance to cold than *P. patens*, which typically inhabits temperate climates (Figure 2). In addition, molecular phylogenetic analysis using the molecular markers *rps4* and *trn*L-F allowed us to identify the phylogenetic position of KMR5045 within the genus *Bryum* (Figure 3). Next, to establish a genetic transfection method for KMR5045, the optimal protoplast isolation conditions and the antibiotic resistance characteristics of this species were determined. The KMR5045 protoplast isolation efficiency was highest when using 1.0% driselase solution dissolved in 0.5 M mannitol (Figure 4B). This enzyme concentration is higher than that of the 0.5% driselase solution used for isolation of *P. patens* protoplasts (Cove et al., 2009a), which may be due to differences in the cell wall compositions of the two species that affect the efficiency of enzyme digestion reactions (Roberts et al., 2012).

Furthermore, the resistance of KMR5045 to various antibiotic concentrations was compared with that of *P. patens* to identify suitable selection conditions for mosses in the Bryaceae family (Figure 5). The sensitivities to antibiotics differed markedly between KMR5045 and *P. patens* in both protonema and protoplast cells. In particular, KMR5045 showed higher sensitivity to kanamycin compared to *P. patens*, implying that the choice of types and concentrations of antibiotics depends on the target species and is critical for the successful genetic transfection of mosses (Figure 6). Since excessive hygromycin can seriously inhibit normal plant development and interfere with the acquisition of transfectants (Yan et al., 2000), it was necessary to select a concentration that is not excessive but effective for selecting transfectants. As can be seen in Figure 6, 28.3% of KMR5045 protoplasts survived at 15 mg L^–1^ hygromycin, and almost all cells died at 20 mg L^–1^ and only 2.5% survived. In addition, the growth of KMR5045 protonema was severely inhibited at 5 mg L^–1^ or higher (Figure 5). Therefore, it was expected that about 70% of untransfected cells could be eliminated at 15 mg⋅L^–1^ hygromycin, and their growth would be severely retarded even if they survived. Therefore, we determined the hygromycin concentration to be 15 mg L^–1^ for the selection of KMR5045 transfectants, and we were able to obtain transfectants successfully in the course of the subsequent experiment.

The PEG-mediated gene transfer method is the most applicable and useful method to transform moss species (Thümmler et al., 1992; Trouiller et al., 2007; Cove et al., 2009b; Nomura et al., 2016). We modified the *P. patens* protocol to achieve genetic transfection of KMR5045 by extending the protoplast regeneration period before antibiotic selection. While *P. patens* generally requires a regeneration period of 5 days (Cove et al., 2009b), the transfection efficiency was found to be highest when KMR5045 protoplasts were transferred to selection medium after 9 days of regeneration (Figure 7). These results indicate that the regeneration time needs to be modified according to the species to be transfected. The entire experimental procedure for the genetic transfection of KMR5045 took 53 days from the first step of protoplast isolation to transfectant selection, and a more detailed timeline is presented in Supplementary Figure 3.

Nearly 16,000 species of mosses vary widely in physiological and metabolic characteristics, but research on these topics is limited (Shaw and Renzaglia, 2004; Laenen et al., 2014). In particular, Arctic mosses have acquired unique physiological traits during their adaptation to their harsh environments (Longton, 1988; Meltofte, 2013; Lewis et al., 2017). In this study, we succeeded in developing axenic cultivation and genetic transfection of KMR5045 belonging to the Bryaceae, which had not been transfected previously. This is the first paper to report the establishment of genetic transfection of an Arctic moss species that survives in an extreme environment. The results of this study will contribute to both basic and applied science fields, such as understanding the mechanism of cold adaptation and the production of useful substances derived from Arctic mosses.

## Data Availability Statement

The original contributions presented in the study are included in the article/Supplementary Materials, further inquiries can be directed to the corresponding author/s.

## Author Contributions

MYB, JL, and HL designed the experiments. MYB, SS, Y-HY performed the experiments. MYB, JL, Y-HY, and HL analyzed the data and wrote the manuscript. All authors have read and agreed to the published version of the manuscript.

## Conflict of Interest

The authors declare that the research was conducted in the absence of any commercial or financial relationships that could be construed as a potential conflict of interest.

## Footnotes

1 https://kvh.kopri.re.kr

## References

[B1] Ahn M. M. Oh T. R. Seo D. H. Kim J. H. Cho N. H. Kim W. T. (2018). Arabidopsis group XIV ubiquitin-conjugating enzymes AtUBC32, AtUBC33, and AtUBC34 play negative roles in drought stress response. *J. Plant Physiol.* 230 73–79. 10.1016/j.jplph.2018.08.010 30193177

[B2] Arroniz-Crespo M. Gwynn-Jones D. Callaghan T. V. Nunez-Olivera E. Martinez-Abaigar J. Horton P. (2011). Impacts of long-term enhanced UV-B radiation on bryophytes in two sub-Arctic heathland sites of contrasting water availability. *Ann. Bot.* 108 557–565. 10.1093/aob/mcr178 21803739 PMC3158694

[B3] Bach S. S. King B. C. Zhan X. Simonsen H. T. Hamberger B. (2014). “Heterologous stable expression of terpenoid biosynthetic genes using the moss *Physcomitrella patens*,” in *Plant Isoprenoids*, ed. Rodríguez-Concepción M. (Berlin: Springer), 257–271. 10.1007/978-1-4939-0606-2_19 24777804

[B4] Byun M. Y. Cho S. M. Lee J. Park H. Lee H. (2019). The complete mitochondrial genome of an Antarctic moss *Chorisodontium aciphyllum* (Hook. f. & Wilson) Broth. *Mitochondrial DNA B* 4 1714–1715. 10.1080/23802359.2019.1605856 PMC9037207 35478861

[B5] Cove D. J. Perroud P. F. Charron A. J. Mcdaniel S. F. Khandelwal A. Quatrano R. S. (2009a). Isolation and regeneration of protoplasts of the moss *Physcomitrella patens*. *Cold Spring Harb. Protoc.* 4:rot5140. 10.1101/pdb.prot5140 20147070

[B6] Cove D. J. Perroud P. F. Charron A. J. Mcdaniel S. F. Khandelwal A. Quatrano R. S. (2009b). Transformation of the moss *Physcomitrella patens* using direct DNA uptake by protoplasts. *Cold Spring Harb. Protoc.* 4:rot5143. 10.1101/pdb.prot5143 20147073

[B7] Cox C. J. Goffinet B. Newton A. E. Shaw A. J. Hedderson T. A. J. (2000). Phylogenetic relationships among the diplolepideous-alternate mosses (Bryidae) inferred from nuclear and chloroplast DNA sequences. *Bryologist* 103 224–241. 10.1639/0007-2745(2000)103[0224:pratda]2.0.co;2

[B8] Duquenne B. Eeckhaut T. Werbrouck S. Van Huylenbroeck J. (2007). Effect of enzyme concentrations on protoplast isolation and protoplast culture of Spathiphyllum and Anthurium. *Plant Cell Tissue Organ Cult.* 91 165–173. 10.1007/s11240-007-9226-3

[B9] Gao B. Zhang D. Li X. Yang H. Zhang Y. Wood A. J. (2015). De novo transcriptome characterization and gene expression profiling of the desiccation tolerant moss *Bryum argenteum* following rehydration. *BMC Genomics* 16:416. 10.1186/s12864-015-1633-y 26016800 PMC4445806

[B10] Greenwood J. L. Stark L. R. Chiquoine L. P. (2019). Effects of rate of drying, life history phase, and ecotype on the ability of the moss *Bryum argenteum* to survive desiccation events and the influence on conservation and selection of material for restoration. *Front. Ecol. Evol.* 7:388.

[B11] Griesbeck O. Baird G. S. Campbell R. E. Zacharias D. A. Tsien R. Y. (2001). Reducing the environmental sensitivity of yellow fluorescent protein. *J. Biol. Chem.* 276 29188–29194. 10.1074/jbc.m102815200 11387331

[B12] Kamisugi Y. Mitsuya S. El-Shami M. Knight C. D. Cuming A. C. Baker A. (2016). Giant peroxisomes in a moss (*Physcomitrella patens*) peroxisomal biogenesis factor 11 mutant. *New Phytol.* 209 576–589. 10.1111/nph.13739 26542980 PMC4738463

[B13] Katoh K. Standley D. M. (2013). MAFFT multiple sequence alignment software version 7: improvements in performance and usability. *Mol. Biol. Evol.* 30 772–780. 10.1093/molbev/mst010 23329690 PMC3603318

[B14] Kostka J. E. Weston D. J. Glass J. B. Lilleskov E. A. Shaw A. J. Turetsky M. R. (2016). The Sphagnum microbiome: new insights from an ancient plant lineage. *New Phytol.* 211 57–64. 10.1111/nph.13993 27173909

[B15] Kumar S. Stecher G. Li M. Knyaz C. Tamura K. (2018). MEGA X: molecular evolutionary genetics analysis across computing platforms. *Mol. Biol. Evol.* 35 1547–1549. 10.1093/molbev/msy096 29722887 PMC5967553

[B16] La Farge C. Williams K. H. England J. H. (2013). Regeneration of little ice age bryophytes emerging from a polar glacier with implications of totipotency in extreme environments. *Proc. Natl. Acad. Sci. U.S.A.* 110 9839–9844. 10.1073/pnas.1304199110 23716658 PMC3683725

[B17] Laenen B. Shaw B. Schneider H. Goffinet B. Paradis E. Desamore A. (2014). Extant diversity of bryophytes emerged from successive post-Mesozoic diversification bursts. *Nat. Commun.* 5:5134. 10.1038/ncomms6134 25346115

[B18] Lang A. S. Kruijer J. D. Stech M. (2014). DNA barcoding of Arctic bryophytes: an example from the moss genus Dicranum (*Dicranaceae, Bryophyta*). *Polar Biol.* 37 1157–1169. 10.1007/s00300-014-1509-7

[B19] Lewis L. R. Ickert-Bond S. M. Biersma E. M. Convey P. Goffinet B. Hassel K. (2017). Future directions and priorities for Arctic bryophyte research. *Arc. Sci.* 3 475–497. 10.1139/as-2016-0043

[B20] Longton R. E. (1988). Adaptations and strategies of polar bryophytes. *Bot. J. Linn. Soc.* 98 253–268. 10.1111/j.1095-8339.1988.tb02429.x

[B21] Medina R. Johnson M. G. Liu Y. Wickett N. J. Shaw A. J. Goffinet B. (2019). Phylogenomic delineation of Physcomitrium (*Bryophyta: Funariaceae*) based on targeted sequencing of nuclear exons and their flanking regions rejects the retention of Physcomitrella. Physcomitridium and Aphanorrhegma. *J. Syst. Evol.* 57 404–417. 10.1111/jse.12516

[B22] Meltofte H. (2013). *Arctic Biodiversity Assessment. Status and Trends in Arctic Biodiversity.* Akureyri: Conservation of Arctic Flora and Fauna.

[B23] Mingeot-Leclercq M. P. Glupczynski Y. Tulkens P. M. (1999). Aminoglycosides: activity and resistance. *Antimicrob. Agents Chemother.* 43 727–737. 10103173 10.1128/aac.43.4.727 PMC89199

[B24] Mueller S. J. Lang D. Hoernstein S. N. Lang E. G. Schuessele C. Schmidt A. (2014). Quantitative analysis of the mitochondrial and plastid proteomes of the moss *Physcomitrella patens* reveals protein macrocompartmentation and microcompartmentation. *Plant Physiol.* 164 2081–2095. 10.1104/pp.114.235754 24515833 PMC3982764

[B25] Nomura T. Sakurai T. Osakabe Y. Osakabe K. Sakakibara H. (2016). Efficient and heritable targeted mutagenesis in mosses using the CRISPR/Cas9 system. *Plant Cell Physiol.* 57 2600–2610. 10.1093/pcp/pcw173 27986915

[B26] Oechel W. C. Sveinbjörnsson B. (1978). “Primary production processes in Arctic bryophytes at barrow, alaska,” in *Vegetation and Production Ecology of an Alaskan Arctic Tundra*, ed. Tieszen L. L. (Berlin: Springer), 269–298. 10.1007/978-1-4612-6307-4_11

[B27] Pandey V. K. Mishra R. Chandra R. (2014). In vitro culture of moss *Bryum coronatum* Schwaegr. (Bryaceae) and it’s phytochemical analysis. *Int. J. Pharm Pharmcol Sci.* 6 307–311.

[B28] Pannewitz S. Green T. G. A. Maysek K. Schlensog M. Seppelt R. Sancho L. G. (2005). Photosynthetic responses of three common mosses from continental Antarctica. *Antarct. Sci.* 17 341–352. 10.1017/s0954102005002774

[B29] Park M. Park H. Lee H. Lee B. H. Lee J. (2018). The complete plastome sequence of an Antarctic bryophyte *Sanionia uncinata* (Hedw.) Loeske. *Int. J. Mol. Sci.* 19:709. 10.3390/ijms19030709 29494552 PMC5877570

[B30] Prestø T. Lüth M. Hassel K. (2014). *Bryophytes of the Longyearbyen Area.* Trondheim: NTNU Vitenskapsmuseet.

[B31] Prigge M. J. Bezanilla M. (2010). Evolutionary crossroads in developmental biology: *Physcomitrella patens*. *Development* 137 3535–3543. 10.1242/dev.049023 20940223

[B32] Rensing S. A. Lang D. Zimmer A. D. Terry A. Salamov A. Shapiro H. (2008). The genome of the moss *Physcomitrella patens* reveals evolutionary insights into the conquest of land by plants. *Science* 319 64–69. 18079367 10.1126/science.1150646

[B33] Reski R. (1998). Development, genetics and molecular biology of mosses. *Botanica Acta* 111 1–15. 10.1111/j.1438-8677.1998.tb00670.x

[B34] Reski R. Bae H. Simonsen H. T. (2018). Physcomitrella patens, a versatile synthetic biology chassis. *Plant Cell Rep.* 37 1409–1417. 10.1007/s00299-018-2293-6 29797047

[B35] Reski R. Parsons J. Decker E. L. (2015). Moss-made pharmaceuticals: from bench to bedside. *Plant Biotechnol. J.* 13 1191–1198. 10.1111/pbi.12401 26011014 PMC4736463

[B36] Roberts A. W. Roberts E. M. Haigler C. H. (2012). Moss cell walls: structure and biosynthesis. *Front. Plant Sci.* 3:166. 10.3389/fpls.2012.00166 PMC3400098 22833752

[B37] Robinson S. A. King D. H. Bramley-Alves J. Waterman M. J. Ashcroft M. B. Wasley J. (2018). Rapid change in East Antarctic terrestrial vegetation in response to regional drying. *Nat. Clim. Chang.* 8 879–884. 10.1038/s41558-018-0280-0

[B38] Roskov Y. Ower G. Orrell T. Nicolson D. Bailly N. Kirk P. (2019). *Species 2000 & ITIS Catalogue of Life [Online].* Available at : www.catalogueoflife.org/annual-checklist/2019 [accessed July 14, 2020]

[B39] Sabovljević A. Sabovljević M. Grubišić D. (2010). Gibberellin influence on the morphogenesis of the moss *Bryum argenteum* Hedw. in in vitro conditions. *Arch. Biol. Sci.* 62 373–380. 10.2298/abs1002373s

[B40] Sabovljević A. Sabovljević M. Grubišić D. Konjević R. (2005). The effect of sugars on development of two moss species (*Bryum argenteum* and *Atrichum undulatum*) during in vitro culture. *Belg. J. Bot.* 138 79–84.

[B41] Schaefer D. Zryd J. P. Knight C. D. Cove D. J. (1991). Stable transformation of the moss *Physcomitrella patens*. *Mol. Gen. Genet.* 226 418–424. 10.1007/bf00260654 2038304

[B42] Schween G. Hohe A. Koprivova A. Reski R. (2003). Effects of nutrients, cell density and culture techniques on protoplast regeneration and early protonema development in a moss. Physcomitrella patens. *J. Plant Physiol.* 160 209–212. 10.1078/0176-1617-00855 12685038

[B43] Shaw J. Renzaglia K. (2004). Phylogeny and diversification of bryophytes. *Am. J. Bot.* 91 1557–1581. 10.3732/ajb.91.10.1557 21652309

[B44] Simmonds J. A. Grainger J. L. (1993). The toxicity of antibiotics to protoplast cultures of *Triticum aestivum* L. *Plant Sci.* 89 209–214. 10.1016/0168-9452(93)90129-n 21802601

[B45] Stanton D. E. Reeb C. (2016). Morphogeometric approaches to non-vascular plants. *Front. Plant Sci.* 7:916. 10.3389/fpls.2016.00916 PMC4921491 27446146

[B46] Street L. E. Subke J. A. Sommerkorn M. Sloan V. Ducrotoy H. Phoenix G. K. (2013). The role of mosses in carbon uptake and partitioning in Arctic vegetation. *New Phytol.* 199 163–175. 10.1111/nph.12285 23614757

[B47] Thümmler F. Schuster H. Bonenberger J. (1992). Expression of oat phyA cDNA in the moss *Ceratodon purpureus*. *Photochem. Photobiol.* 56 771–776. 10.1111/j.1751-1097.1992.tb02233.x 1475324

[B48] Trouiller B. Charlot F. Choinard S. Schaefer D. G. Nogue F. (2007). Comparison of gene targeting efficiencies in two mosses suggests that it is a conserved feature of Bryophyte transformation. *Biotechnol. Lett.* 29 1591–1598. 10.1007/s10529-007-9423-5 17565445

[B49] Wagner T. A. Cove D. J. Sack F. D. (1997). A positively gravitropic mutant mirrors the wild-type protonemal response in the moss *Ceratodon purpureus*. *Planta* 202 149–154. 10.1007/s004250050113 11541791

[B50] Walker D. A. Daniels F. J. A. Alsos I. Bhatt U. S. Breen A. L. Buchhorn M. (2016). Circumpolar Arctic vegetation: a hierarchic review and roadmap toward an internationally consistent approach to survey, archive and classify tundra plot data. *Environ. Res. Lett.* 11:055005 10.1088/1748-9326/11/5/055005

[B51] Wandrey F. Henes B. Zülli F. Reski R. (2018). Biotechnologically produced moss active improves skin resilience. *SOFW J.* 144 34–37.

[B52] Yan B. Reddy M. S. Collins G. B. Dinkins R. D. (2000). *Agrobacterium tumefaciens*–mediated transformation of soybean [Glycine max (L.) Merrill.] using immature zygotic cotyledon explants. *Plant Cell Rep.* 19 1090–1097. 10.1007/s002990000236 30754775

[B53] Yu G. Cheng Q. Xie Z. Xu B. Huang B. Zhao B. (2017). An efficient protocol for perennial ryegrass mesophyll protoplast isolation and transformation, and its application on interaction study between LpNOL and LpNYC1. *Plant Methods* 13:46. 10.1186/s13007-017-0196-0 PMC5460552 28592987

