# Supplementary material for: Transfection of Arctic Bryum sp. KMR5045 as a Model for Genetic Engineering of Cold-Tolerant Mosses
Source: Front Plant Sci. 2021 Jan 8;11:609847. doi: 10.3389/fpls.2020.609847 (PMC7873996; doi:10.3389/fpls.2020.609847)
Supplement: Supplementary file 1 [file Table_1.DOCX]

Supplementary Materials


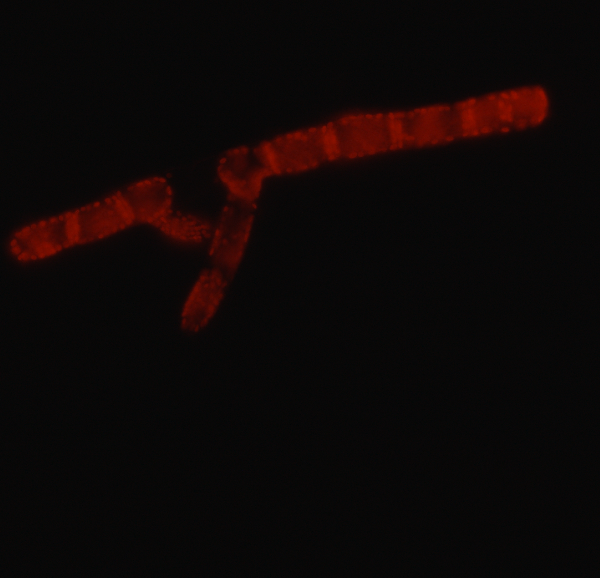

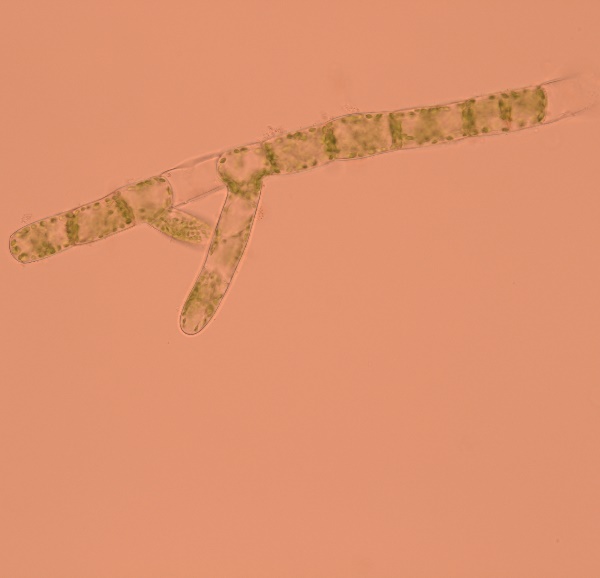


Bright field

Chlorophyll

**Supplementary Figure 1.** Morphological feature of cells in the protonema stage of KMR5045. Arrows indicate cells containing less developed chloroplasts. Bottom image of chlorophyll fluorescence shows the absence of chloroplast in cells. Scale bars; 20 μm.


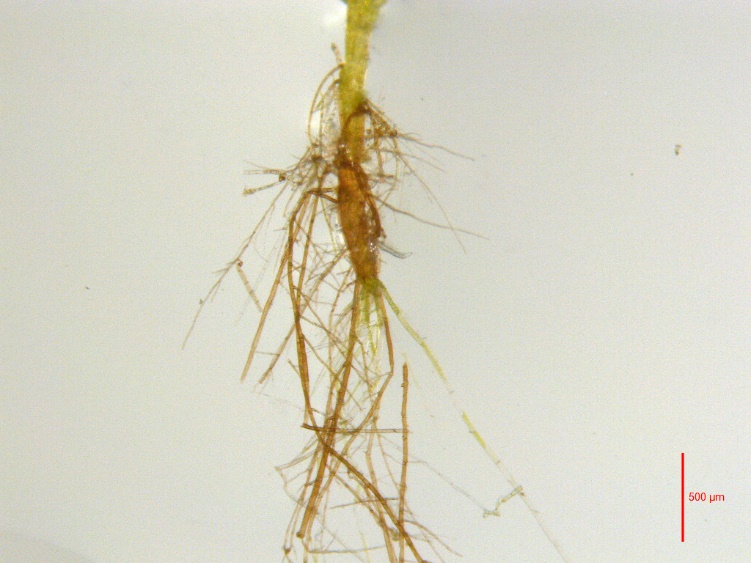


**Supplementary Figure 2.** Morphological feature of rhizoids in the gametophore stage of KMR5045. Arrows indicate points of rhizoid branching. Scale bars; 100 μm.

Protoplast isolation and PEG-mediated transfection

Transfer to regeneration medium

Day 01

Day 02

Selection of transfectants

Day 53

Transfer to antibiotics free medium

Day 25

Transfer to the 2^nd^ selection medium

Day 39

Transfer to the 1^st^ selection medium

Day 11

**Supplementary Figure 3.** Timeline of the protocol for the genetic transfection of KMR5045

| **Supplementary Table 1.** List of synthetic oligonucleotide sequences used in this study. | | |
| --- | --- | --- |
| Oligonucleotide name | Sequence | Purpose |
| rps4 F | ATGTCCCGTTATCGAGGACCT | Molecular marker for species identification |
| rps4 R | TACCGAGGGTTCGAATC | Molecular marker for species identification |
| trnL F | CGGAATTGGTAGACGCTACG | Molecular marker for species identification |
| trnF R | ATTTGAACTGGTGACACGAG | Molecular marker for species identification |
| Hpt F | TGGAACTGACAGAACCGCAA | Genomic DNA PCR |
| Hpt R | GCGGGAGATGCAATAGGTCA | Genomic DNA PCR |
| Tubulin F | ATCACGGAGTTCCAGACCAA | Genomic DNA PCR and RT-PCR |
| Tubulin R | CCACTGCCGCATTCACAT | Genomic DNA PCR and RT-PCR |
| Citrine F | ATGGGAGGTGGAGGTGGAGC | RT-PCR |
| Citrine R | CTAGGCCCCAGCGGCCGCAG | RT-PCR |

| **Supplementary Table 2.** List of taxa used in phylogenetic analysis, with GenBank accession number. | | | | |  |
| --- | --- | --- | --- | --- | --- |
| **Order** | **Family** | **Species** | ***rps4*** | ***trn*L-F** | |
| Bryales | Bryaceae | KMR5045 |  |  | |
|  |  | *Bryum wrightii* | AY078330 | AY078303 | |
|  |  | *Bryum pallens* | AF521688 | AY150356 | |
|  |  | *Bryum cyclophyllum* | AF521684 | AY150352 | |
|  |  | *Bryum demissum* | AY078334 | AY078307 | |
|  |  | *Rhodobryum giganteum* | AF023789 | AF023737 | |
| Bartramiales | Bartramiaceae | *Philonotis fontana* | AF023801 | AF023758 | |
| Orthotrichales | Orthotrichaceae | *Orthotrichum lyellii* | AF023814 | AF023727 | |
| Hypnales | Pylaisiaceae | *Calliergonella lindbergii* | AF143035 | AF161128 | |
|  | Fontinalaceae | *Fontinalis antipyretica* | AF023817 | AF023771 | |
| Hypnodendrales | Racopilaceae | *Racopilum tomentosum* | AJ251314 | AF215904 | |
|  | Hypnodendraceae | *Bescherellia brevifolia* | AJ251313 | AF215903 | |
|  |  | *Mniodendron dendroides* | AF023822 | AF023746 | |
| Splachnales | Meesiaceae | *Leptobryum pyriforme* | AF023802 | AF023736 | |
|  | Splachnaceae | *Splachnum ampullaceum* | AJ251308 | AF215899 | |
| Pottiales | Pottiaceae | *Syntrichia ruralis* | AF023831 | AF023722 | |
| Encalytales | Encalytaceae | *Encalypta rhaptocarpa* | AF023777 | AF023717 | |
| Diphysciales | Diphysciaceae | *Diphyscium foliosum* | AF223034 | AF229891 | |
| Timmiales | Timmiaceae | *Timmia sibirica* | AF023775 | AF023715 | |
| Funariales | Funariaceae | *Funaria hygrometrica* | AF023776 | AF023716 | |
| Polytrichales | Polytrichaceae | *Polytrichum commune* | AF208428 | AF231907 | |
| Dicranales | Dicranaceae | *Dicranum scoparium* | AF234158 | AF234159 | |
| Tetraphidales | Tetraphidaceae | *Tetraphis pellucida* | AF231896 | AF231908 | |
| Sphagnales | Sphagnaceae | *Sphagnum palustre* | AF231892 | AF231902 | |
| Takakiales | Takakiaceae | *Takakia lepidozioides* | AF231894 | AF231904 | |
| Marchantiales | Marchantiaceae | *Marchantia polymorpha* | JX241481 | KX792409 | |
